# Assessment of the Dynamic Exposure to PM_2.5_ Based on Hourly Cell Phone Location and Land Use Regression Model in Beijing

**DOI:** 10.3390/ijerph18115884

**Published:** 2021-05-30

**Authors:** Junli Liu, Panli Cai, Jin Dong, Junshun Wang, Runkui Li, Xianfeng Song

**Affiliations:** 1 College of Resources and Environment, University of Chinese Academy of Sciences, Beijing 100049, China; liujunli16@mails.ucas.ac.cn (J.L.); caipanli19@mails.ucas.ac.cn (P.C.); dongjin19@mails.ucas.ac.cn (J.D.); wangjunshun20@mails.ucas.ac.cn (J.W.); xfsong@ucas.ac.cn (X.S.); 2 State Key Laboratory of Resources and Environmental Information System, Institute of Geographic Sciences and Natural Resources Research, Chinese Academy of Sciences, Beijing 100101, China; 3 Center for Ocean Mega-Research of Science, Chinese Academy of Sciences, Beijing 100101, China; 4 Sino-Danish College, University of Chinese Academy of Sciences, Beijing 100049, China; 5 Sino-Danish Educational and Research Centre, University of Chinese Academy of Sciences, Beijing 100190, China

**Keywords:** cell phone, activity pattern, exposure assessment, fine particulate matter, land use regression model

## Abstract

The spatiotemporal locations of large populations are difficult to clearly characterize using traditional exposure assessment, mainly due to their complicated daily intraurban activities. This study aimed to extract hourly locations for the total population of Beijing based on cell phone data and assess their dynamic exposure to ambient PM_2.5_. The locations of residents were located by the cellular base stations that were keeping in contact with their cell phones. The diurnal activity pattern of the total population was investigated through the dynamic spatial distribution of all of the cell phones. The outdoor PM_2.5_ concentration was predicted in detail using a land use regression (LUR) model. The hourly PM_2.5_ map was overlapped with the hourly distribution of people for dynamic PM_2.5_ exposure estimation. For the mobile-derived total population, the mean level of PM_2.5_ exposure was 89.5 μg/m^3^ during the period from 2013 to 2015, which was higher than that reported for the census population (87.9 μg/m^3^). The hourly activity pattern showed that more than 10% of the total population commuted into the center of Beijing (e.g., the 5th ring road) during the daytime. On average, the PM_2.5_ concentration at workplaces was generally higher than in residential areas. The dynamic PM_2.5_ exposure pattern also varied with seasons. This study exhibited the strengths of mobile location in deriving the daily spatiotemporal activity patterns of the population in a megacity. This technology would refine future exposure assessment, including either small group cohort studies or city-level large population assessments.

## 1. Introduction

Detailed exposure assessment is the basis of air pollution-related epidemiological studies [1]. The accuracy of the estimated exposure will affect the derived exposure–response relationship [2] and lead to different decisions. Therefore, accurately characterizing the spatiotemporal location of people and the corresponding pollution concentration are two fundamental procedures in exposure assessment. 

However, the location of people and the concentration of pollution are always varying with time and are difficult to describe in detail. The geographic locations of people are changing due to human activities, mostly in a home-office, home-schooling, or in other daily patterns [3]. The outdoor air pollution concentration changes spatially along the travel route, with higher concentrations near traffic roads or industry settings and lower concentrations farther away from emission sources [4]. The air pollution concentration at a given location may show a daily cycle overlapped with seasonal trends [5]. When people go through different places, they would expose to the location-time specified on-spot air pollution concentration, and the exposure accumulates during this spatiotemporal activity process [6,7].

In practical use, there have been several methods used to quantify air pollution exposure in populations of different sizes. Small population exposure studies would use a personal sampler to directly collect the time-space accumulated exposure [8,9,10,11] or would record the trip through a positioning device at the same time [12,13]. A large population panel study might collect the home addresses or zip codes of residents and the monitored air pollution from sparse fixed sites for approximation [14,15,16], while for city-level health risk assessment, a total population and the average concentration of monitoring sites are commonly used [17,18,19]. The real location of people, their daily commute inside the city, and the representativeness of monitoring sites are not of concern.

Problems remain in using static population and fixed monitoring sites as surrogates for the actual exposures of populations [1]. The discrepancy between the two parties would stem from the high spatial heterogeneity of air pollutants and the biased setting of monitoring sites, combined with an inaccurate measure of the spatial distribution of the population. Even when a population density map based on census data was used [20], the distribution of people could still not be accurately accounted for. A large portion of the population registers in one district but actually lives in another district, for work, school, or other reasons. The separation of officially registered and actual residences is currently very common in big cities in China, thus resulting in a special phenomenon where census population data cannot reflect the actual living arrangements [21]. Therefore, it has been a great challenge to quantify the location of city-level populations and the air pollutant concentrations they are being exposed to.

With the increasingly widespread use of cell phones, base station networks have emerged as a good method for locating large populations [22]. The cellular network infrastructure were quickly developed with almost worldwide coverage [22], and cellular towers were set up densely in Chinese cities during the past two decades. The spatial location of a mobile client could be recorded by base stations when they were moving through the network. As a result, quantifying the diurnal activity pattern of the total population based on such positioning technology becomes promising and practical. For example, Deville et al. (2014) used log-linear regression to estimate the relationship between nighttime mobile phone-call records and the census population. Their study showed the potential of mobile phone data to estimate daily, weekly, and seasonal population dynamics [23]. Liu et al. (2018) proposed an approach for population dynamics mapping based on the time series of individual trajectories that were reconstructed from mobile phone records. The proposed method is effective at estimating the population distribution, and it also has potential for use in mapping population dynamics at fine spatial and temporal resolutions even when the users’ location information is intermittent or discontinuous [24]. Population activity patterns derived from mobile devices have been used to evaluate population-weighted exposure to air pollution and show significantly different results from traditional methods in New York City [25].

In addition, the land use regression model (LUR) has been widely used in air pollution modeling and exposure assessment [26,27]. Air pollutants vary largely across space in the urban area; however, the monitoring sites are commonly sparse, and it is hard to capture the short-distance spatial variation of the air pollutant concentrations among sites. Therefore, taking advantage of spatially detailed geographic information, LUR generally shows higher accuracy in air pollution modeling when compared to spatial interpolation methods based on spatial autocorrelation (such as inverse distance-weighted and kriging) [28,29].

The objective of this study was to investigate the detailed exposure of the total population to ambient PM_2.5_ in Beijing. The diurnal dynamics of the population spatial distribution were derived from hourly records of base station networks, and a fine resolution PM_2.5_ map was produced by the land use regression model. The hourly adjusted PM_2.5_ maps were overlapped with the hourly population distribution to finally obtain the city-average outdoor exposure.

## 2. Materials and Methods

### 2.1. Cellular Positioning

Cellular service areas are divided into cells that are centered with a base station (i.e., a cellular tower). When a user connects to the cellular network through a phone call, a message, or 4G internet, the mobile device is allocated to the base station with the strongest signal [22]. Then, the base station and phone identification are recorded in the cellular network system. The spatial location of the base station is used to represent the position of the cell phone under contact. The location accuracy of this cellular positioning is mainly dependent upon the spatial density of the base stations, a higher density of base stations will divide the space into smaller cell sizes and increase the positioning accuracy.

### 2.2. Study Area and Site Description

**Figure 1:**
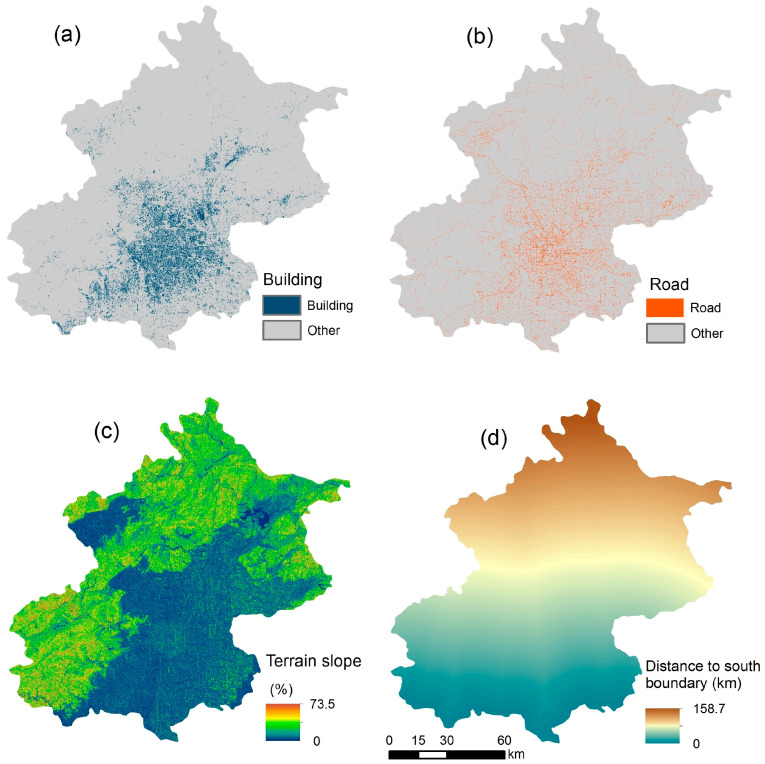
Some of the prepared land use variables. (**a**) building; (**b**) major road; (**c**) terrain slope; and (**d**) distance to the south boundary.

The entire Beijing municipality was included in this study, which covered an area of about 16,410 km^2^ and a total population of about 21.7 million at the end of 2015. The urban area is mainly concentrated in the plain area in the central and southern parts of Beijing, the city center is represented by several major ring roads, and a few scattered urban areas are located within the surrounding districts. The northern and western parts are mountainous rural areas that are mostly covered by forests and hold much fewer people (Figure 1). 

The geographic locations of more than 15,000 base stations, together with the hourly number of cell phones that contacted each base station, were collected under the 4G network of Beijing. The data was provided by a communications operating company through project cooperation and is not publicly available at present. Continuous phone counts were obtained on a work day and a weekend day of 2015 and were integrated into 24 h. Hourly PM_2.5_ data from 1 January 2013 to 31 December 2015 were collected at 35 fixed monitoring sites from the real-time Air Quality System (AQS) of the Beijing Municipal Ecological and Environmental Monitoring Center (http://zx.bjmemc.com.cn/getAqiList.shtml, accessed on 29 May 2021). Some outlier observations (e.g., >1000 μg/m^3^) were dropped. The data cleaning, combined with equipment errors, resulted in the loss of less than 10% of the hourly observations in total. Many geographic variables potentially correlate with PM_2.5_, including both natural and human-related variables. Land use variables related to geographical location, traffic pollution, city construction, terrain, and vegetation were prepared for the construction of the LUR model. Population density from NASA (GPWv4) [30] was collected to compare with the exposure result of cell phone locations. Road data were obtained from Amap. Building and NDVI variables were extracted or calculated from Landsat-8 OLI images. ASTER GDEM was used to derive the terrain slope. The Euclidean distance of each raster grid to the south boundary of Beijing was calculated in ArcGIS 10.6. All the variables were prepared in a spatial resolution of 30 m × 30 m. The original population density map at 1 km × 1 km resolution was resampled to 30 m × 30 m by the nearest neighbor approach to match other variables.

### 2.3. LUR Model for PM_2.5_ Predictions

The relationship between PM_2.5_ and the land use variable within the neighborhood area (buffer size) varies with the neighborhood scale. To provide a sufficient amount of buffers, a distance-decay buffer searching technique was used. Based on this technique, it is easier to find the optimal buffer size [31,32]. The buffer size with the highest correlation coefficient between PM_2.5_ and the land use variable was derived, and variables at their optimal buffer sizes were selected to build the land use regression model through a step-wise regression analysis. 

Consistent with our previous studies [33], the aerosol optical depth (AOD), the distance to south boundary, the distance to the nearest major road, the road density, the population density, the construction area ratio, NDVI, and the terrain slope were prepared for the LUR model in this study (Figure 1). The constructed area in Beijing is mainly concentrated in the central-south area. A large portion of Beijing is mountainous and appears in the west and north. The collinearity between the above variables was considered. To select the final land use variables from all of the prepared variables for the LUR model building, a stepwise linear regression was conducted. When selecting regression predictors, each variable was examined to determine whether or not it would be entered into the regression function based on its improvement of the regression. The significance level (*p* value) of a variable for its entry or removal was set at 0.05 and 0.1, respectively. The final LUR model was established by using only the entered land use variables. The overall regression models, considering both the spatial trend and local variation, were used to estimate PM_2.5_ concentrations at a spatial resolution of 30 m × 30 m. Models for different seasons were also separately built using the above variables and methods. 

To obtain the hourly PM_2.5_ map, the diurnal pattern of PM_2.5_ in 24 h was derived based on the hourly observations. Because LUR models performed better at longer time periods, we only built yearly and seasonal models. We calculated the yearly average and the seasonal average PM_2.5_ in 24 h based on the hourly observations from 2013 to 2015 to derive the diurnal pattern within a day. The diurnal pattern of PM_2.5_ in 24 h at a yearly or seasonal time scale was later used to adjust the predicted daily average PM_2.5_ maps to derive hourly spatial distributions.

Model performance was evaluated by several indices, both in the modeling period and the validation period. Model R^2^, adjusted model R^2^, leave-one-out cross-validation (CV) R^2^, mean absolute error (MAE), and root mean square error (RMSE) were used to describe model accuracy.

### 2.4. Exposure Assessment

The spatial distribution of cell phones at each hour was used to represent the corresponding hourly population distribution. The location of the base station was overlapped with the hourly map of PM_2.5_, and the hourly PM_2.5_ concentrations at each base station were extracted. The station-specific concentration was then weighted by its population (i.e., cell phones) to obtain the city-level average exposure. In addition, the much-detailed time-space activity pattern of people was related to the corresponding hourly PM_2.5_ for exposure assessment. As a result, a more detailed assessment was obtained compared to other methods, such as the average concentration of monitoring sites or exposure based on the traditional census population density map.

The hourly population density maps, which represented the daily commute of residents in Beijing, were overlaid with the same yearly or seasonal PM_2.5_ map to assess the variation of exposure solely caused by commute.

Different exposure assessment methods were also compared. Several commonly used outdoor exposure measures, such as using the observations from the U.S. Embassy in Beijing, the mean concentration of the 35 sites, directly using the mean concentration of LUR map, PM_2.5_ weighted by the census population, and PM_2.5_ weighted by the active population (this study) were implemented. The statistics were completed in ArcGIS and MS Excel.

## 3. Results

### 3.1. Spatial Distribution of Base Stations and Average PM_2.5_

LUR models were established for seasonal and yearly average PM_2.5_ concentrations (Table 1). In each model, two or three predictors were finally entered as predicting land use variables, with one representing the regional trend (distance to the south boundary of Beijing, DTS) and other variables accounting for local variations. All of the models performed excellent according to the adjusted model R^2^ and the leave-one-out cross-validation (CV) R^2^. Specifically, the LUR performed better for the yearly average and winter models. Accuracy for the summer model was lower, but the R^2^ was still higher than 0.7.

The error indices, such as the MAE and RMSE, also showed high accuracy for the derived LUR models (Table 2). The models achieved low error amounts across each of the four seasons, with MAE and RMSE values less than 10 μg/m^3^. Errors in the winter model were slightly higher, but the mean concentration during that time was also much higher than in other seasons. The LUR models, during the model building and cross-validation periods as well as the models for different seasons, all showed high stability and accuracy.

**Figure 2:**
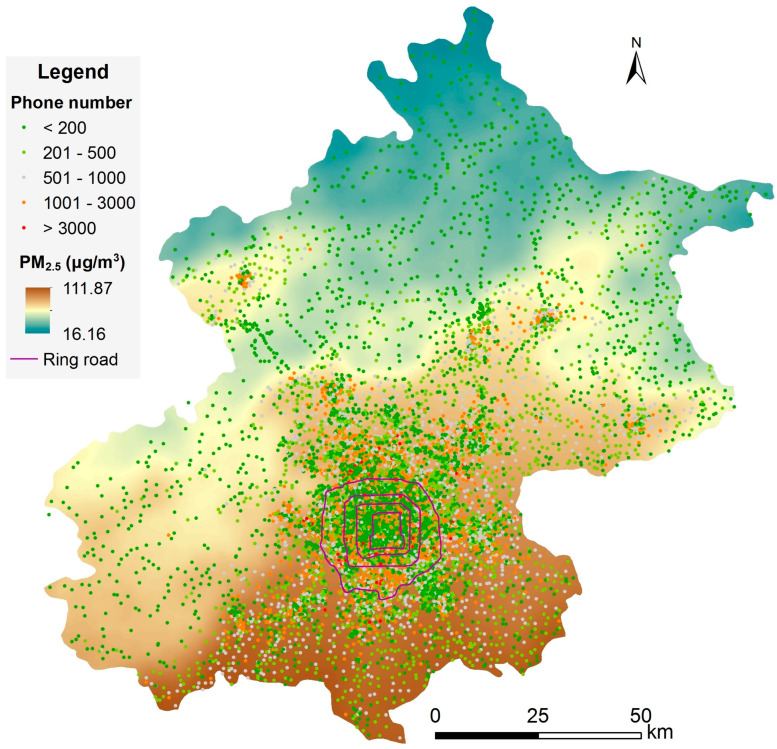
Average PM_2.5_ concentration map and base station distribution, with the cell phone number of each base station at 15:00.

The average PM_2.5_ map, derived from the LUR model, was overlapped with all the base stations (Figure 2). PM_2.5_ was generally higher in the south part and decreased gradually while moving to the north. Lower PM_2.5_ concentrations could be observed in the northern mountainous areas. Local high concentrations could be observed in the populous towns. The base stations were heterogeneously distributed, with a higher density in urban areas and a much lower density in rural areas. The cell phone number at each base station also varied obviously across space, with the busiest base stations in the center of the city or town. 

However, the spatial distribution of the cell phone numbers (related to population density) was not fully consistent with PM_2.5_ concentration. Higher PM_2.5_ concentrations appeared in the south and east parts of Beijing, but the density of cell phone numbers was much lower than in the central urban area. Most people in Beijing were living in the urban area with a moderate PM_2.5_ concentration of this region.

### 3.2. Commute of People

In order to investigate the daily commute of people inside Beijing, we calculated the ratio of the total population moving into each ringed road during the daytime. We compared the distribution of the population at 15:00 (representing daytime at work) to that of 2:00 (representing nighttime at home) (Table 3). In total, 10.8% of the total population commuted from outside of the 5th into the 5th ringed road region (667.8 km^2^) during work time, and back to their homes at night; this was the net portion of people commuting across the rings. Considering opposite-direction-commute and the people commuting inside a ring, the daily commuting population would be much larger than this number. 

### 3.3. Diurnal Pattern of PM_2.5_

**Figure 3:**
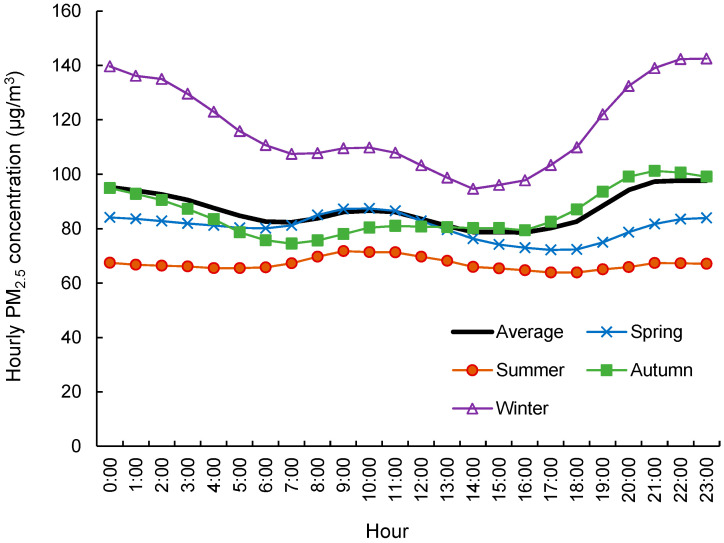
Diurnal change of PM_2.5_ in different seasons.

PM_2.5_ concentrations showed a strong diurnal pattern across 24 h in Beijing (Figure 3). The diurnal peaks were roughly affected by an increase in human activity in the morning and a reduction due to dispersion at late night. The patterns and concentration levels varied largely for different seasons. A much higher concentration level was observed in the winter, with its PM_2.5_ concentration over 140 μg/m^3^ at night, which demonstrated the varied exposure levels for different seasons. Diurnal patterns in winter and autumn showed a rapid increase from afternoon to night. However, the peak during rush hour in the morning during the summer was more obvious than in other seasons, and the rise at night in the summer was insignificant.

Based on the hourly concentrations in Figure 3, we calculated the hourly adjustment factor for each season by dividing the corresponding hourly concentration by the daily mean concentration. The seasonal LUR PM_2.5_ map was then adjusted by the hourly factor to get a PM_2.5_ map for each hour. This process was also applied to the yearly average LUR PM_2.5_ map.

### 3.4. Exposure Assessment Based on the Active Population

Due to the spatial-temporal variation of PM_2.5_ concentrations, the exposed PM_2.5_ concentration of a person changed with either the spatial location or time. In order to investigate the variation of exposure that was caused only by the commute of the population during a day (i.e., spatial effect), the average PM_2.5_ concentration map was overlapped with each of the hourly population maps. Thus, the hourly variation of exposure was solely attributed to commute. Moreover, the hourly PM_2.5_ concentration map was also overlapped with the corresponding hourly population map to derive the hourly exposure. In this way, the joint effect of commute and diurnal PM_2.5_ variation was considered. 

**Figure 4:**
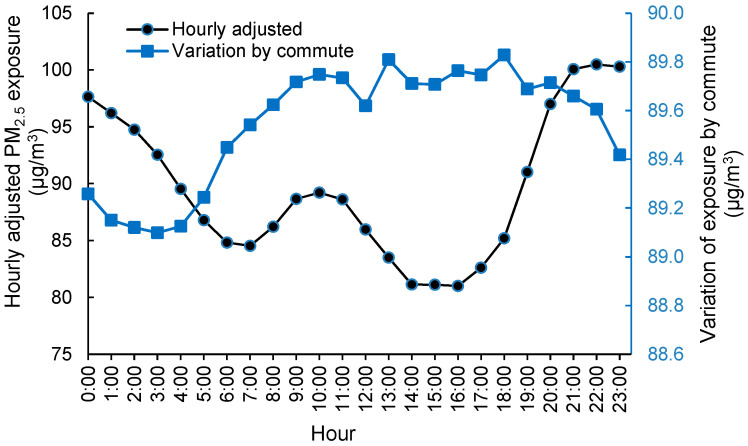
Average hourly variation of PM_2.5_ exposure from 2013 to 2015. The blue line represented the hourly variation solely caused by the commute of the population, and the black line represented the total variation caused by both the commute of the population and the hourly change of PM_2.5_ concentrations.

The hourly exposure for the total population based on the above two methods was calculated, and its diurnal variations were assessed (Figure 4). An increase in the exposure level due to population commute could be observed in the morning from 4:00 to 10:00 when people were going to work, whereas the decrease in exposure lasted from 18:00 to 3:00 when people were gradually returning home. The increase in exposure in the morning and the decrease at night indicated that people were exposed to higher PM_2.5_ concentrations at work addresses than at home addresses. There were also two sudden increases in exposure at 13:00 and 18:00 when most people were on their way to dinner or were going home. Such activities were usually close to traffic roads and probably resulted in elevated PM_2.5_ exposure.

Furthermore, the exposure of the commuting population under hourly, varying PM_2.5_ concentrations was explored. The combined exposure, including the spatial and temporal variations of people and PM_2.5_, was designated as actual exposure (Figure 4). The final exposure varied significantly across 24 h, with generally lower levels in the daytime and higher levels in the nighttime. The diurnal pattern was similar to that of the daily PM_2.5_ pattern (Figure 3). The different variation magnitudes of the two curves revealed that the temporal change of PM_2.5_ concentration contributed more hourly variations to the total exposure on an hourly scale than did the commute of people. This was reasonable because the diurnal variation of PM_2.5_ was larger than the spatial variation of PM_2.5_, as most people were concentrating in the urban areas where PM_2.5_ levels were moderate in Beijing.

**Figure 5:**
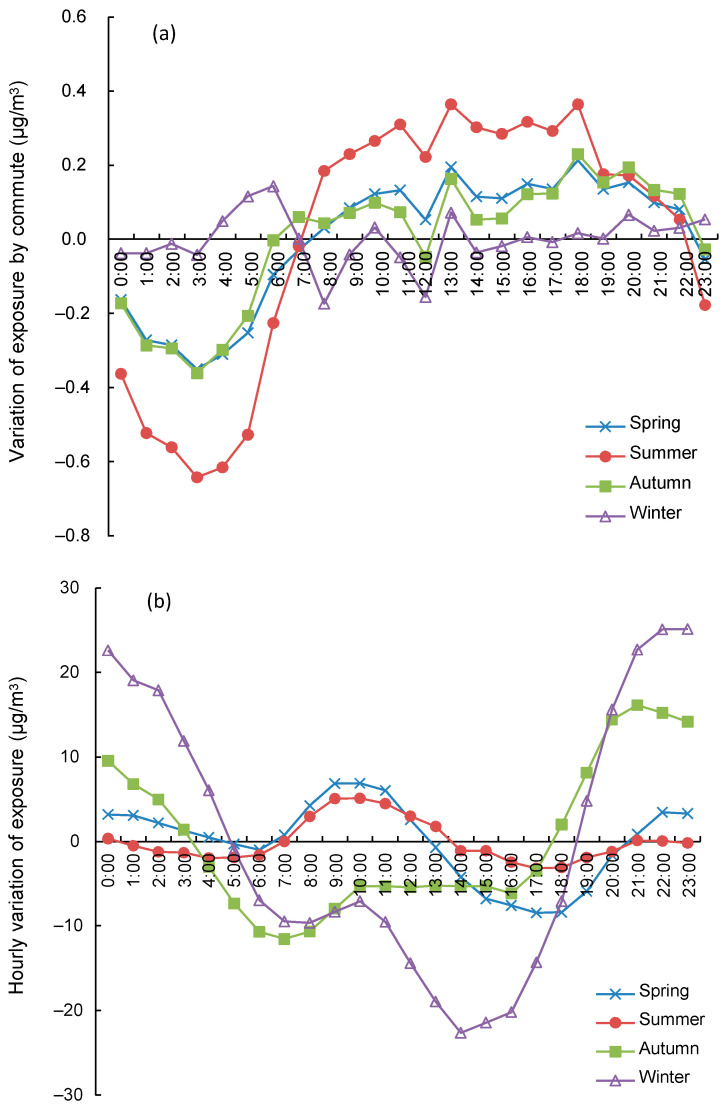
Hourly variation of PM_2.5_ exposure for different seasons after subtracting their mean values. (**a**) the hourly variation of exposure solely caused by the commute of the population, (**b**) the hourly total variation of exposure caused by both the commute of population and the hourly change of PM_2.5_ concentration.

Similarly, the hourly exposure of the commuting population across the four seasons was also assessed (Figure 5). As shown in Figure 5a, the variation of exposure caused by commuting across 24 h during different seasons showed different patterns. Commuting during the summer showed greater hourly variation, with higher PM_2.5_ exposure at working addresses and lower exposure at home addresses. In contrast, hourly exposure during the winter was seldomly affected by commuting. This could be attributed to the high similarity of PM_2.5_ concentrations in the urban areas during the winter in Beijing. In general, the variation in the total-population exposure that was caused by commuting was small and less than 2 μg/m^3^.

Figure 5b showed the variation in hourly exposure for different seasons. Dominated by meteorological conditions, such as temperature, wind speed, and mixing height, hourly exposure variations were larger for the different seasons compared to those caused by commuting. Hourly variations were larger in winter and autumn than in spring and summer. 

**Figure 6:**
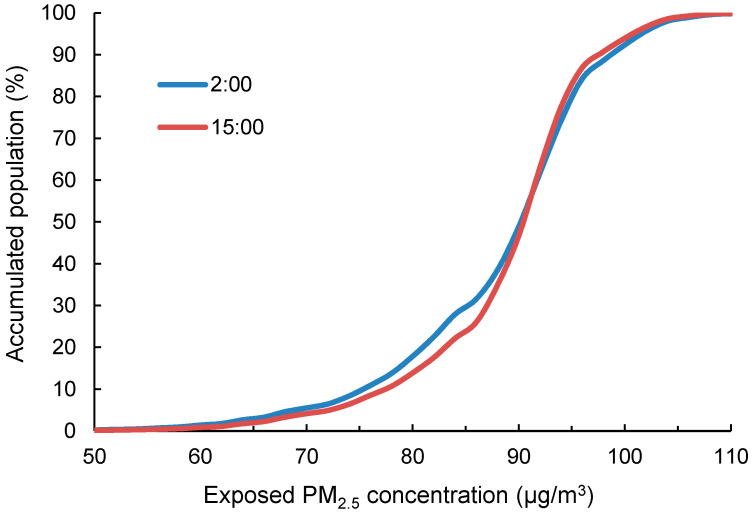
The change in the exposure curve due to the commute of the population. The line at 2:00 represents people at home addresses, and the line at 15:00 represents the working addresses after commuting.

The effect of population commute on exposure was further investigated by comparing the population exposure curves before and after accounting for population commute (Figure 6). The two exposure curves showed that more people were exposed to lower PM_2.5_ (<90 μg/m^3^) at 2:00 at night when most people were at home. The two accumulated curves crossed at about 90 μg/m^3^, which referred to the central urban area and demonstrated the increase in the number of people in the urban areas during the daytime at 15:00. The differences between the two curves revealed the change of the accumulated exposure under each PM_2.5_ level that was caused by the daily commute of the population.

### 3.5. Different Exposure Assessment Methods

Several commonly used outdoor exposure measures were utilized to compare the exposure in this study that was based on mobile location (Table 4). Using only the one site from the U.S. Embassy in Beijing, the urban area showed a higher level of exposure at 93.5 μg/m^3^. While the mean of the LUR map, including the entire study area, was 68.3 μg/m^3^, which was much lower due to the large proportion of the less-populated mountainous areas (Figure 2). The mean of the 35 monitoring sites was 87.2 μg/m^3^, which was close to the census population density-weighted mean of 87.9 μg/m^3^. The mobile population-weighted mean was 89.5 μg/m^3^, which was slightly higher than that of the census population. The reason for this might be that the census was based on home addresses and did not consider the daily commute of the population, whereas higher exposure during the daytime due to commuting could be captured by cell phone locations.

The results from different measures of exposure also indicated that the population was not uniformly distributed in Beijing. Most people were in urban areas where the PM_2.5_ concentration was higher than in the northern rural areas. However, the distribution of the monitoring sites in Beijing captured the exposure level very well. It can be found in Table 4 that the mean of the 35 sites was close to the population density-weighted mean PM_2.5_ concentration. One possible reason may be that most monitoring sites were installed in towns to represent the average condition of air pollution around such areas. 

## 4. Discussion

This study estimated the average exposure of the population in Beijing to outdoor PM_2.5_ based on hourly cell phone locations. Compared to previous studies in China or other countries, the seasonal and yearly LUR models established in this study were all excellent [27,34,35,36,37,38]. The low error rate and high R^2^ during model fitting and the cross-validation period showed the success of our modeling approach as well as the high stability and distinguished prediction capability of the LUR in the Beijing area. 

The daily activity pattern of people showed that more than 10% of the total population commuted into the center of Beijing, which was mainly within the 5th ringed road, during the daytime. PM_2.5_ concentrations at home addresses were generally lower than at work addresses, as work addresses were usually in urban areas and were close to traffic roads. Commuting in the summer caused greater hourly exposure variation, but hourly exposure in the winter was seldomly affected by commuting. The city mean exposure level for the population was 89.5 μg/m^3^ from 2013 to 2015, which was about 9 times that of the WHO air quality guideline of 10 μg/m^3^ for the annual mean [39].

The city-level hourly variation of exposure that was contributed by people’s commuting was small in Beijing (Figure 4 and Figure 5a). This could be mainly explained by the spatial living patterns of people and the spatial variation of PM_2.5_ concentrations. A large portion of people were living in urban areas (Figure 2). Only the commuting people who were living in suburban or rural areas and working in urban areas contributed to the change in total exposure (Table 3). Although the difference in PM_2.5_ concentrations between home addresses and work addresses were up to 20 or 30 μg/m^3^ for some commuting people, such difference would be much smaller for most people. At the same time, the differences in exposure introduced by the commuting population (at least 10.8%) was finally averaged out by the total population. Another reason is the balance effect of people commuting between the south and north areas. Because of the clear regional decreasing trend of PM_2.5_ from the south to the north (Table 1 and Figure 2) in Beijing, the effect of commuting on city-level exposure would be largely cancelled by people commuting from the south to the north versus the north to the south. The people from regions having varied PM_2.5_ concentrations, along with both coming to the central urban area or to each other’s area, would cancel out the effect of commuting on the variation of exposure. 

The daily change of the spatial patterns of the population and exposure in this study was not as significant as that in New York City (NYC) [25]. The main reason would be the different city structure of Beijing compared to NYC. Beijing, spatially, is much more balanced in residential and commercial regions. In each district, the residential and commercial regions are mixed or alternately distributed. However, the midtown to downtown regions of NYC are mainly commercial regions, with crowds of people during the daytime but much fewer residents at night. Therefore, the net commuting portion of people during the daytime in Beijing would be less than that of NYC.

This study showed the strengths of cell phone-related big data in deriving spatiotemporal population activity patterns of a megacity of over 21 million people. The technology would facilitate future exposure assessments, including either small group cohort studies or city-level large population studies. Cell phone locations from the base station would cover almost the full population, rather than using the Global Positioning System (GPS) module in a smartphone. The GPS method was only effective for users who were opening their GPS modules and were using a location recording software [6,7,9].

However, several limitations in this study should be noted. Firstly, the total amount of cell phones connected to all of the base stations changed remarkably during a day, from about 5 million to 18 million. That fewer phones were connected at night was probably due to the powering off or discontinued movement of cell phones. Therefore, the hourly number of cell phones was standardized when deriving the hourly spatial map of the population. The relationship between the population and the number of phones would change by time and region, which was not considered in this study. Secondly, building height was not considered in this study. People in buildings at different vertical layers were exposed to PM_2.5_ concentrations that differed from those at the ground level. Thirdly, the location of the base station was adopted to represent the cell phones under connection, and the specific location of each person was not further geocoded in detail. The approximation of individual location by the location of the base station might introduce positioning errors of individual cell phones at a level of hundreds of meters, and this error also varied from urban to rural areas. People were usually distributed around the base stations, and errors from individual exposure due to inaccurate locations would be partly balanced out when averaging the concentrations around the base stations. However, the accuracy of the estimated exposure would be reduced by the incompatible spatial resolution between the individual location and the PM_2.5_ map.

The study findings provide insightful information for the spatiotemporal population activity patterns in Beijing. The methods in this study also help to refine exposure assessment in air pollution and health effect studies. This study provides information for other cities in allocating monitoring sites for air pollution monitoring, exposure assessment, and health effect investigation campaigns. The future integration of multi-source data, including various sources of positioning data, air pollution measurements, and human behavior data, would be beneficial for urban environmental and epidemiological studies.

## Figures and Tables

**Table 1:** LUR models for seasonal PM_2.5_ concentration.

Prediction Function ^a^	Adjusted R^2^	CV R^2^
Spring = 62.45 − 0.20 × DTS2190m − 22.96 × NDVI60m + 60.67 × YearlyAOD1500m	0.86	0.83
Summer = 70.30 − 0.22 × DTS4020m + 49.25 × Road5010m	0.77	0.74
Autumn = 115.71 − 0.50 × DTS1890m − 1.26 × Slope3840m	0.85	0.84
Winter = 22.90 + 323.41 × WinterAOD990m − 0.410 × DTS2370m	0.89	0.86
Average = 115.83 − 0.48 × DTS2400m − 1.15 × Slope4620m	0.89	0.87

^a^ LUR models for the average PM_2.5_ concentration during each season from 2013 to 2015 (μg/m^3^); DTS2190m is the distance to the south boundary of Beijing with a buffer size of 2190 m, and the unit of this distance is km; NDVI60m is the Normalized Difference Vegetation Index with a buffer size of 60 m; YearlyAOD1500m is the yearly average AOD with a buffer size of 1500 m; Road5010m is the road area ratio with a buffer size of 5010 m; Slope4620m is the terrain slope with a buffer size of 4620 m; and the unit of the slope is percent (%).

**Table 2:** Error indices for LUR models for the different seasons during the modeling period and the cross-validation period.

Season	Mean (μg/m^3^)	Modeling (μg/m^3^)		Cross-Validation (μg/m^3^)	
		MAE	RMSE	MAE	RMSE
Spring	80.60	2.52	3.15	2.84	3.55
Summer	67.03	2.87	3.63	3.13	3.97
Autumn	85.74	4.25	5.25	4.65	5.67
Winter	117.31	6.30	8.30	7.03	9.63
Average	87.42	3.37	4.33	3.72	4.75

**Table 3:** Proportion of the total population commuted into each ringed road in the central urban area during the daytime.

Ringed Road	Net increase during Daytime (Percent of Total Population)
2nd	2.9
3rd	6.4
4th	9.7
5th	10.8
Outside 5th	−10.8

**Table 4:** Different measures of outdoor PM_2.5_ exposure.

Exposure Method	Mean of the U.S. Embassy	Mean of the 35 Sites	Mean of the PM_2.5_ Map	Population Density-Weighted	Mobile Population-Weighted
Exposed PM_2.5_ (μg/m^3^)	93.5	87.2	68.3	87.9	89.5

## Data Availability

Data sharing is not applicable to this article.
